# Bacterial Solutions to the Problem of Sex

**DOI:** 10.1371/journal.pbio.0050245

**Published:** 2007-09-18

**Authors:** J. Arjan G. M de Visser

## Abstract

Why have sex? It seems that genetic studies in the normally non-sexual*E. coli* may have the key.

“Why sex?” is a question that has occupied the minds of evolutionary biologists for more than a century. The evolution of organisms that mix their genes during reproduction is considered one of the major transitions in evolution [1], because it fundamentally changed how genes are transmitted to the next generation. In asexual reproduction, offspring inherit a more or less unaltered genome from the parent. In sexual reproduction, genetic material is first reduced in the gametes (sperm or ovaries) and then fused with that of another individual, before new offspring may develop. Sexual reproduction has led to the evolution of males and females with different and sometimes even opposing reproductive interests and behavior. The consequence of sex, which many evolutionary biologists think explains its existence, is the production of genetic variation by mixing genes from different individuals.

Nineteenth-century German biologist August Weismann [2] was the first to realize that sex can produce variation and that variation allows organisms to better respond to selection. Three models have since been proposed as general explanations for the advantages of sex by elaborating Weismann's idea: (i) sex facilitates adaptation to a novel environment by combining beneficial gene variants, or alleles, from different genomes (Fisher-Muller (FM) model [3,4]); (ii) sex helps organisms stay adapted to existing environmental conditions by combining and efficiently removing deleterious alleles (mutational deterministic (MD) model [5]); or—a combination of these two ideas—(iii) sex helps to liberate beneficial alleles from linked deleterious alleles in the genome in which they arose (mutational load (ML) model [6,7]).

Several recent laboratory evolution experiments have found support for Weismann's proposal that sex facilitates the response to selection [8]. However, distinguishing between the different models remains a challenge. In particular, it has been difficult to alter recombination without changing other variables that might confer a sex advantage, such as environmental conditions or non-neutral genetic markers. In one attempt to overcome these problems, Rice and Chippindale [7] smartly used genetic tools to construct synthetic chromosomes of the fruit fly Drosophila melanogaster that could not recombine and hence evolved asexually. This trick allowed the authors to study the effect of recombination on the fate of an introduced beneficial mutation without the obscuring effects of differences in initial fitness or environmental conditions used to induce sex in some populations. In another recent attempt, Goddard et al. [9] constructed asexual yeast strains by deleting genes involved in the production of gametes. This also allowed them and others [10] to compare the evolution of sexual and asexual yeast populations under the same conditions. However, none of these studies has been able to relate observed fitness changes to the genetic changes responsible, a connection that provides a useful handle for distinguishing between the relative contributions of beneficial and deleterious mutations to any observed sex advantage, and hence for distinguishing among the three competing models explaining sex.

In this issue of *PLoS Biology*, Tim Cooper [11] takes the art of testing sex hypotheses to a new level of experimental rigor. Instead of using constructed asexual variants of a sexual species, Cooper used Escherichia coli—which, like all bacteria, reproduce asexually—to test the relative contribution of the models mentioned above. The choice of E. coli may seem odd but is an excellent one for several reasons. First, by introducing the F plasmid carrying the genes for conjugation (the bacterial equivalent of sex), Cooper could study the consequence of sex—i.e., genetic recombination—while excluding obscuring effects from changed conditions or fitness. Second, by manipulating the mutation rate, he could create conditions where competition among different beneficial mutations is known to limit the rate of adaptation [12], a prerequisite for the advantage of recombination according to the FM model. Third, by using the same strains and conditions that have been used in a previous long-term evolution experiment [13], Cooper knew which beneficial mutations were to be expected and could study the dynamics of a major adaptive mutation in order to distinguish between the FM and ML models.

The results provide clear support for the FM model. After 1,000 generations of laboratory evolution, all lines showed greater fitness—refuting the effect of deleterious mutations (MD model) as primary explanation, and recombining (rec+) lines (that is, those with sex) had improved more than nonrecombining (rec−) lines. To distinguish between the explanations offered by the FM model (sex combines beneficial mutations) and the ML model (sex liberates beneficial mutations from deleterious genetic backgrounds), Cooper looked at the competitive dynamics of a focal beneficial mutation (in regulatory gene *spoT*), which was found in rec+ and rec− lines alike. This allowed him to test two specific predictions of the FM model. First, competition with other clones carrying beneficial mutations should slow down the fixation of the focal mutation in rec− lines, but not in rec+ lines. Second, this same interference should lower the fitness of clones carrying the focal mutation relative to contemporary clones in rec− lines, but not in rec+ lines, once competing clones reach appreciable frequencies (Figure 1). As a control for the possible effects of deleterious mutations (and hence the ML model), the fitness of these clones relative to their ancestors should not decrease during the same time interval. Both predictions were supported by the data. By using a simple experimental system, which allowed an unprecedented level of control of evolutionary variables and detailed information on the genetics and population dynamics underlying the evolutionary changes, this study provides the most specific support found so far for the FM model.

**Figure 1:**
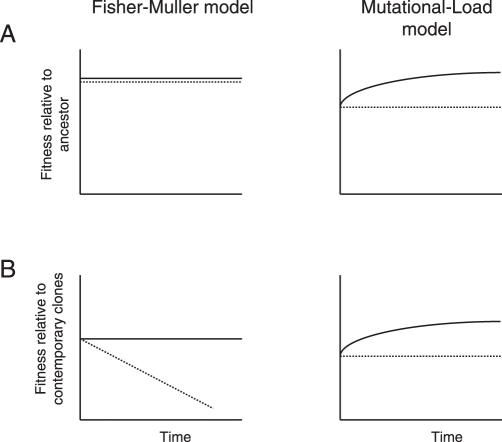
Predictions from the FM and ML Models about the Fitness of a Focal Beneficial Mutation during Its Increase in Frequency in the Population The advantage of recombination, according to the FM model, derives from combining beneficial mutations; the advantage predicted by the ML model results from the liberation of the focal beneficial mutation from linked deleterious mutations in the genome in which it originated. (A) Fitness of the focal beneficial mutation in a recombining (rec+, solid line) and nonrecombining (rec−, dashed line) population measured relative to the ancestor. (B) Fitness of the same mutation relative to that of contemporary clones from the same population. It is assumed that the focal mutant in the rec− populations does not accumulate further beneficial mutations.

How strongly do these data reject alternate explanations for the evolutionary maintenance of sex? Parasites or otherwise-fluctuating environmental conditions can be ruled out from the experimental conditions set during evolution. Similarly, the faster removal of deleterious mutations, proposed by the MD model, is unlikely to have been very important, because both of its prerequisites—a rate of deleterious mutations of at least one per genome per generation and prevailing synergistic interactions among mutations—are not met by E. coli. However, it is more difficult to refute any effect from deleterious mutations, because they are likely also the most frequent type of mutations in these bacterial populations. Although they are not the primary explanation, sex benefits from liberating occasional beneficial mutations from linked deleterious mutations (ML model) and from the more efficient removal of multiple deleterious mutations (MD model) may have contributed to the higher fitness of the rec+ populations. Another complicating factor is that the support for the FM model based on the dynamics of the focal mutation depends on the assumption that beneficial mutations go to fixation one-by-one in the rec− populations. A recent study of asexual adaptation in yeast [14] indicates that when beneficial mutations are sufficiently common to cause clonal interference, additional beneficial mutations may occur on the background of a focal beneficial mutation that will then collectively go to fixation. Although in such a scenario clonal interference will still occur—only some of it will be between clones carrying multiple beneficial mutations—it will alter the competitive dynamics of beneficial mutations in sexual and asexual populations in ways yet to be explored [15]. A systematic comparison over time of the fitness of clones carrying *spoT* mutations from rec− and rec+ populations relative to the ancestor and to contemporary clones (along the lines suggested in Figure 1) would be one way to see whether and how recombination affects adaptation when multiple beneficial mutations go to fixation together.

How relevant are these bacterial results for understanding the evolutionary maintenance of sex in higher organisms? In other words, does the bacterial support for the FM model mean that recombination between beneficial mutations is the universally best explanation for the existence of sex and recombination? Not necessarily. The benefits of sex depend on factors that are likely to differ among species, including the variability of environmental conditions, the population size, and the rate, fitness effects, and interactions of mutations. We are gathering information on some of these factors for micro-organisms, but they are hardly known for higher organisms. Only the patient collection of such data from a variety of different organisms may ultimately reveal whether organisms share general unifying principles that allow a universal explanation for sex, or alternatively whether different explanations are required for different species [16]. Possibly unifying principles may be found in shared genetic architectures. For instance, the way genes interact to cause the structures and functions necessary for survival and reproduction is one source of evolutionary constraint that allows sex to be beneficial [8,17].

Undoubtedly, the study by Cooper [11] will stimulate further studies of the evolution of recombination using simple experimental systems. Only by manipulating single evolutionary variables at a time, while removing the confounding effects of other variables, can the contribution of the different models explaining sex be tested in unambiguous ways. To reach the desired level of experimental control, the subject of study must be isolated from its natural environment and stripped from all its nonessential attributes. While such reductionism holds the danger of changing the “natural behavior” of the subject of study, it is arguably the most efficient way forward. Most importantly, as the study by Cooper shows, simple systems benefit from the detailed information they can provide on the evolutionary dynamics of genetic change and how this differs between sexual and asexual populations. One can only hope that new genetic technologies will soon allow similar experimental rigor in studies with other organisms.

## Abbreviations

FM: Fisher–Muller
MD: mutational deterministic
ML: mutational load
